# The effect of stellate ganglion block on postoperative recovery quality in peri-menopausal women undergoing gynecological laparoscopic surgery

**DOI:** 10.3389/fmed.2025.1561512

**Published:** 2025-06-09

**Authors:** Xueling Yuan, Yijie Tang, Xiyuan Xie, Jiapeng Qiu, Yupeng Han, Peng Ke, Chengjie Zheng, Kai Zeng, Xiaodan Wu

**Affiliations:** ^1^Shengli Clinical Medical College of Fujian Medical University, Department of Anesthesiology, Fujian Provincial Hospital, Fuzhou, Fujian, China; ^2^Fuzhou University Affiliated Provincial Hospital, Department of Anesthesiology, School of Medicine, Fuzhou University, Fuzhou, Fujian, China; ^3^Department of Anesthesiology, The First Affiliated Hospital of Fujian Medical University, Fuzhou, China

**Keywords:** ultrasound-guided stellate ganglion block, postoperative recovery quality, peri-menopausal, gynecological laparoscopic surgery, gynecology and obstetrics

## Abstract

**Objective:**

This study aims to observe whether right-sided stellate ganglion block (SGB) before surgery can improve early postoperative recovery quality in peri-menopausal women undergoing elective gynecological laparoscopic surgery.

**Methods:**

Ninety-four peri-menopausal women, who meet the inclusion criteria, scheduled for gynecological laparoscopic surgery were selected (Trial Registration: ChiCTR2200057907, March 21, 2022). They were randomly assigned into two groups (Group S and Group C). Group S received right-sided SGB under ultrasound guidance, combined with tracheal intubation and general anesthesia, with an injection of 4 mL of 0.2% ropivacaine. Group C underwent only ultrasound scanning combined with tracheal intubation and general anesthesia. The primary outcome was postoperative recovery quality at 24 h, assessed using the 40-item Quality of Recovery (QoR-40) questionnaire. Secondary outcomes included: (1) Heart rate (HR) and Mean Arterial Pressure (MAP); (2) Resting pain scores; (3) Recovery of gastrointestinal function postoperatively; (4) Postoperative adverse reactions within 24 h.

**Results:**

At 24 h postoperative, Group S had a higher QoR-40 total score compared to Group C with a corrected mean difference of 12.50. Significant differences in HR were noted at T3, T5, and T6, and in MAP at T2, T4, T6, and T7. The resting pain scores at 4, 8, and 12 h postoperatively differed significantly between the two groups. (4) Compared with Group C, Group S had a shorter time to first flatus and a shorter time to the first return of bowel sounds. The incidence of postoperative abdominal distension was lower in Group S compared to the Group C. (5) The incidence of postoperative nausea, vomiting, headache, shoulder pain, and throat pain was lower in Group S than Group C, with statistically significant differences.

**Conclusion:**

Preoperative single-session stellate ganglion block improves 24-h postoperative recovery in peri-menopausal women undergoing gynecological laparoscopic surgery by alleviating pain, stabilizing hemodynamics, promoting gastrointestinal recovery, and reducing postoperative adverse reactions.

**Clinical trial registration:**

www.chictr.org.cn, identifier ChiCTR2200057907.

## Key summary points

**Why carry out this study?**

This study was conducted to investigate whether preoperative right-sided stellate ganglion block (SGB) can improve early postoperative recovery in peri-menopausal women undergoing elective gynecological laparoscopic surgery. The aim was to assess whether SGB could enhance recovery quality by reducing postoperative pain, stabilizing hemodynamics, and promoting gastrointestinal recovery, potentially offering a better recovery experience for these patients.

**What was learned from the study?**

1. **Improved Postoperative Recovery:** The group receiving SGB (Group S) showed a significantly higher quality of recovery (QoR-40 total score) at 24 h postoperative compared to the control group (Group C).
2. **Hemodynamic Stability:** Group S exhibited significant differences in heart rate (HR) and mean arterial pressure (MAP) at various time points, suggesting better hemodynamic stability post-surgery.
3. **Reduced Postoperative Pain:** Resting pain scores were significantly lower in Group S at several postoperative time points (4, 8, and 12 h), indicating that SGB helped alleviate pain more effectively.
4. **Faster Gastrointestinal Recovery:** Group S had a shorter time to the first flatus and the first return of bowel sounds, indicating improved gastrointestinal recovery.
5. **Reduced Postoperative Adverse Reactions:** The incidence of postoperative adverse reactions, including nausea, vomiting, headache, shoulder pain, and throat pain, was significantly lower in Group S compared to Group C, suggesting that SGB helped reduce common postoperative discomforts.

## 1 Introduction

The World Health Organization (WHO) defines perimenopause as the permanent cessation of menses and decreased levels of ovarian steroid hormones (estrogen and progesterone) due to loss of ovarian follicles. Perimenopause usually occurs around the age of 50 years and is marked by a change in the length of two adjacent menstrual cycles which is more than 7 days within 10 months and a period of amenorrhea of less than 12 months (1, 2). The perimenopausal women may experience symptoms such as hot flashes, night sweats, insomnia, vaginal dryness, and emotional disturbance (3, 4).

At present, laparoscopic surgery has become the main type of gynecological surgery. Compared with open surgery, laparoscopic surgery has the advantages of reducing postoperative pain, shortening the length of hospital stay, reducing bleeding, reducing complications, reducing the length of hospital stay and total cost (5). However, CO_2_ can cause increased sympathetic nerve activity and hemodynamic fluctuations during endoscopic surgery (6). And the residual CO_2_ can irritate the phrenic nerve and cause pain in the shoulder and upper abdomen (7). The above factors seriously affect the recovery status of perimenopausal women. Therefore, there is an urgent need to explore therapeutic measures to improve the quality of recovery after gynecological laparoscopic surgery in perimenopausal patients.

Stellate ganglion block (SGB) is the injection of local anesthetics around the stellate ganglion deep in the prevertebral fascia to block the sympathetic nerve activity dominated by the stellate ganglion (8), which can reduce the excitability of sympathetic nerve, make intraoperative hemodynamics more stable (9), and improve postoperative sleep (10). It also can alleviate the symptoms of hot flashes and improve sleep quality in perimenopausal and breast cancer patients (11–14). However, among the existing investigations correlating with SGB, there is a lack of evidence of the effect of SGB on improving the quality of recovery after gynecological laparoscopic surgery in perimenopausal women.

Therefore, this study aims to investigate the effect of preoperative ultrasound-guided SGB on the recovery quality of perimenopausal patients after gynecological laparoscopic surgery, so as to provide an applicable clinical method for accelerating the postoperative recovery of perimenopausal patients.

## 2 Materials and methods

### 2.1 Study design and participants

A prospective, randomized controlled, double-blind clinical trial, approved by the Ethics Committee of Fujian Provincial Hospital (K2021-12-064, December 30, 2021) and written informed consent was obtained from all subjects participating in the trial. The trial was registered in the Chinese Clinical Trial Center^1^ (ChiCTR2200057907, March 21, 2022), which was conducted at Fujian Provincial Hospital from March 2022 to January 2023. The study protocol was performed in accordance with the Declaration of Helsinki. The female patients aged 40-60 years with ASA I-II who were consistent with perimenopausal syndrome and scheduled for laparoscopic surgery were included in this study. The exclusion criteria included BMI ≥ 30 kg/m^2^, incompatibility, difficult airway, allergic to anesthetics, abnormal coagulation disorders, anatomical deformities of the neck or shoulders, severe disease and taking painkillers. All participants gave their informed consent.

### 2.2 Grouping and blinding

All the patients were divided into two groups by random number generator: receiving ultrasound-guided stellate ganglion block (Group S) and just an ultrasound scan (Group C). The allocation ratio was 1:1, and the group assignments were then sealed in opaque envelopes. The anesthesiologists, surgeons, nurses, data collectors, and data analysts were unaware of the group assignments, except for the doctor in charge of the nerve block.

### 2.3 Ultrasound-guided stellate ganglion block

**FIGURE 1:**
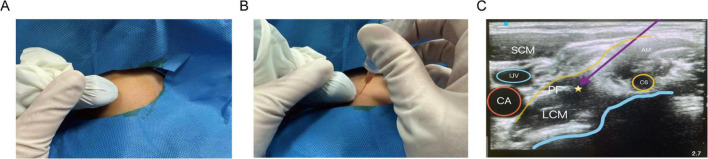
Ultrasound-guided stellate ganglion block images. **(A)** Position of the ultrasound probe and the neck; **(B)** Direction of the ultrasound probe; **(C)** In-plane ultrasound technique showing the stellate ganglion block area and its surrounding structures. AM, Anterior scalene muscle; SCM, Sternocleidomastoid muscle; C6, Sixth cervical nerve root; PF, Prevertebral fascia; LCM, Longus colli muscle; ⋆, Stellate ganglion block area; ↘, Simulated puncture path; IJV, Internal jugular vein.

SGB was performed in the anesthesia recovery room 15 min before anesthesia induction. The procedure was conducted by the same anesthesiologist skilled in ultrasound-guided SGB techniques. Ultrasound-guided right-sided SGB and right stellate ganglion ultrasound scans were performed on the enrolled patients. The SGB procedure followed the method described by Hai-Hua Shan et al. (15). The detailed steps were as follows: The patient was positioned supine without a pillow, with the head turned 45° to the left. The mouth was slightly open, and the anterior neck muscles were relaxed. After routine skin disinfection and draping, a high-frequency linear transducer (6–13 MHz) was used. The probe was aligned parallel to the cricoid cartilage plane and angled at 45° to the sagittal plane of the neck, moving from the medial edge of the sternocleidomastoid muscle outward (Figures 1A,B). Ultrasound clearly displayed the transverse process of the C6 vertebra and its anterior and posterior tubercles. The probe was then slightly moved toward the C7 vertebra until the anterior tubercle of the C6 transverse process disappeared from the ultrasound screen. The operator carefully identified the anatomical structures, including the C6 nerve root, carotid artery, jugular vein, vertebral artery, thyroid gland, esophagus, and trachea. Using color Doppler imaging, the vascular distribution along the puncture path was evaluated. Using an in-plane technique, the needle was inserted through the gap between the C6 nerve root and the internal jugular vein. When the needle tip reached the surface of the longus colli muscle beneath the anterior fascia (Figure 1C), aspiration confirmed the absence of blood, air, or cerebrospinal fluid. In the S group, 4 mL of 0.2% ropivacaine was slowly injected while observing the spread of the anesthetic. Successful block was indicated by the appearance of Horner’s syndrome manifestations on the injection side, including ptosis, conjunctival hyperemia, ipsilateral pupil constriction, facial flushing, anhidrosis, and nasal congestion.

### 2.4 Anesthesia method

#### 2.4.1 Preparation before induction

Patients fasted for 6 h and refrained from drinking for 2 h before surgery, with no preoperative medication administered. After entering the operating room, a peripheral venous line was established on the right side, and sodium-potassium-magnesium-calcium glucose injection was appropriately supplemented. Standard monitoring was connected, including electrocardiogram (ECG), oxygen saturation, non-invasive blood pressure, and Bispectral Index (BIS). Fifteen minutes before anesthesia induction, an experienced anesthesiologist performed ultrasound-guided right-sided SGB and stellate ganglion ultrasound scanning. In the S group, 4 mL of 0.2% ropivacaine was slowly injected, while in the C group, only ultrasound scanning at the same site was conducted.

#### 2.4.2 Anesthesia induction and maintenance

Oxygen was administered via a mask at a flow rate of 6 L/min, and patients were instructed to breathe calmly for 3-5 min to ensure sufficient preoxygenation and nitrogen washout. Intravenous induction drugs were then administered: midazolam 0.02 mg/kg, sufentanil 0.6 μg/kg, propofol 1.5-2.0 mg/kg, and rocuronium 0.6 mg/kg. Once the BIS value reached 40-50 and complete muscle relaxation was achieved, an appropriately sized wire-reinforced endotracheal tube (typically size 6.5 for female patients) was inserted. The cuff was inflated and secured, with cuff pressure maintained at 25 cmH_2_O. Mechanical ventilation was initiated with the following settings: oxygen flow rate 2 L/min, inspired oxygen concentration 50%, tidal volume 6-8 mL/kg, respiratory rate 10–15 breaths/min, and an inspiration-to-expiration ratio (I:E) of 1:2. Ventilator parameters were adjusted based on intraoperative end-tidal carbon dioxide (PetCO_2_) to maintain PetCO_2_ at 35-45 mmHg.

Total intravenous anesthesia (TIVA) was used intraoperatively, with continuous micro-pump infusion of propofol at 40-160 μg/kg/min and remifentanil at 0.15-0.4 μg/kg/min, maintaining BIS values between 40 and 60. Dosages were adjusted according to BIS values and patient blood pressure. Rocuronium was supplemented as needed. Circulatory stability was maintained throughout surgery. Hypotension (blood pressure 20% below baseline) was addressed by identifying and correcting the cause, with ephedrine administered if necessary. Hypertension (blood pressure 20% above baseline) was managed with intravenous urapidil. Bradycardia (heart rate < 50 bpm) was treated with intravenous atropine, while tachycardia (heart rate > 100 bpm) was managed with esmolol after ruling out causes such as shallow anesthesia or hypovolemia. Propofol and remifentanil infusions were discontinued during pneumoperitoneum closure and skin suturing. Postoperatively, the tracheal tube was removed after the BIS value exceeded 80, and the patient awakened naturally. If muscle relaxation recovery was inadequate, neostigmine 1 mg and atropine 0.5 mg were administered to reverse it, provided no contraindications were present. The patient was then transferred to the anesthesia recovery room for observation with nasal oxygen (2 L/min) for at least 30 min. After stabilization of heart rate and blood pressure, the patient was returned to the ward.

#### 2.4.3 Postoperative analgesia

Patients received pain relief through PCIA (Patient-Controlled Intravenous Analgesia) after surgery. The analgesic pump formula consisted of sufentanil 2 μg/kg, flurbiprofen axetil 200 mg, and vitamin B6 200 mg, diluted with normal saline to a total volume of 100 mL. The PCIA pump was configured as follows: an initial dose of 2 mL, maintenance dose of 2 mL, single additional dose of 2 mL, lockout time of 15 min, and a maximum dose of 15 mL/h. PCIA could be used continuously for 48 h. Patients and their families were instructed on how to use the intravenous analgesic pump. If the patient experienced severe postoperative pain (VAS ≥ 4), they were advised to press the PCA button for pain relief. Pain levels were reassessed 10 min after pressing. If severe pain persisted, the ward doctor could provide tramadol for oral analgesia.

### 2.5 Observation indicators

#### 2.5.1 Baseline Information

Basic patient information, including age, height, weight, BMI, ASA classification, type of surgery, duration of surgery, time with the endotracheal tube, and duration of anesthesia.

#### 2.5.2 Primary Observation Indicators

The QoR-40 (Quality of Recovery-40) scale score at 24 h postoperatively. The QoR-40 includes 5 major domains with a total of 40 items, ranging from 40 (minimum) to 200 (maximum). The domains include: Physical comfort (12 items), Pain (7 items), Psychological support (7 items), Emotional state (9 items), Ability for self-care (5 items). Follow-up personnel recorded the scores for each question based on the patient’s condition and calculated the total preoperative and 24-h postoperative scores for each patient. Secondary Observation Indicators: HR and MAP at the following time points: Before SGB (T0), 15 min after SGB (T1), immediately before intubation (T2), immediately after intubation (T3), immediately during pneumoperitoneum (T4), at the end of surgery (T5), immediately after extubation (T6), 30 min after extubation (T7). Postoperative pain assessment:Using the VAS (Visual Analog Scale) to evaluate resting pain at 1, 4, 8, 12, and 24 h postoperatively. VAS scoring: 0 = no pain, 10 = severe pain; 1-3 = mild pain, 4-6 = moderate pain, 7-10 = severe pain. If VAS ≥ 4, patients were instructed to press the PCA button for analgesia. The total number of PCA presses and the analgesic pump medication usage within 24 h postoperatively were recorded. Recovery of gastrointestinal function: Time to return of bowel sounds, time to first flatus, presence of postoperative abdominal distension. Incidence of postoperative adverse reactions:Including nausea, vomiting, headache, shoulder pain, and sore throat.

### 2.6 Statistical analysis

According to a review of relevant literature (16), a difference of 10 points or more in the QoR-40 score indicates a clinically significant improvement in postoperative recovery quality. In a preliminary pilot study conducted earlier in this project, the control group (*n* = 20) had a QoR-40 score of 171.3 ± 14.65 at 24 h post-surgery. Assuming a significance level of α = 0.05 and a statistical power of β = 0.8, the sample size was calculated using PASS 15 software. Considering a 20% dropout rate, the final calculated sample size is 47 patients per group, with a total of 94 patients included in this study.

This study conducted statistical analysis based on the ITT (Intention-to-Treat) principle, including all randomized patients. Continuous variables: Normality was tested using the Shapiro-Wilk test and Q-Q plots. Data conforming to a normal distribution were expressed as mean ± standard deviation ( ± SD) and analyzed using a t-test or one-way analysis of variance (ANOVA). Data with a non-normal distribution were expressed as median (interquartile range) [M (IQR)] and compared using the Mann-Whitney *U*-test. Categorical variables: Presented as counts or percentages (n or [%]) and compared using the chi-square test or Fisher’s exact test. Postoperative QoR-40 scores at 24 h: Analyzed using analysis of covariance (ANCOVA), with preoperative QoR-40 scores used as covariates for adjustment. Repeated measures data (HR, MAP, VAS): Analyzed using a linear mixed-effects model, treating individual patients as random effects and group, time, and their interaction as fixed effects. A random intercept and an unstructured covariance structure were applied. This study primarily focused on the effects of interventions:

If no interaction effect was observed (*P*_*time*^*group_ > 0.05), the main effects of the interventions were analyzed. If interaction effects were present (*P*_*time*^*group_ < 0.05), the effects of interventions at specific time points were analyzed. Statistical analyses and graphical representations were performed using SPSS 22.0 and GraphPad Prism 8 software. A *P*-value < 0.05 was considered statistically significant.

## 3 Results

### 3.1 Patient characteristics

**FIGURE 2:**
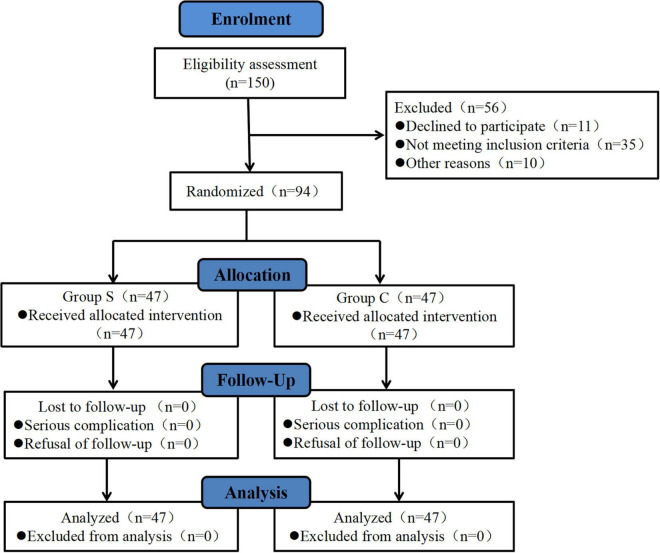
CONSORT diagram of study flow.

A total of 150 patients were enrolled from March 2022 to January 2023. Fifty-six patients were excluded, including 11 patients who refused to participate in the study, 35 patients who did not meet the inclusion criteria, and 10 patients whose surgery were canceled. Finally, 94 patients were enrolled in the study and randomly assigned to the Group C or the Group S. The CONSORT flow chart for all participants is shown in Figure 2. As shown in Table 1, there was no significant difference in age, BMI, anesthesia duration, operation duration, tube duration, ASA classification, preoperative diagnosis, type of surgery, and preoperative QoR-40 score between the two groups (*p* > 0.05), and the baseline demographic data were balanced and comparable.

**TABLE 1:** Patient demographic data.

	Group S (*n* = 47)	Group C (*n* = 47)	*P-*value
Age (years), [IQR]	51 [49, 53]	50 [48, 52]	0.071^β^
BMI (kg/m^2^), mean ± sd	24.96 ± 2.53	24.51 ± 2.69	0.414^α^
Anesthesia duration (min), [IQR]	132 [120, 160]	130 [120, 146]	0.756^β^
Operation duration (min), [IQR]	123 [108, 140]	120 [105, 127]	0.366^β^
Tube duration (min), [IQR]	140 [125, 168]	136 [129, 150]	0.791^β^
**ASA**			
I	32	29	0.517^γ^
II	15	18	
**Preoperative diagnosis, n (%)**			
Uterine fibroids	14 (29.78%)	17 (36.17%)	0.669^γ^
Multiple uterine fibroids	13 (27.66%)	17 (36.17%)	
Adenomyosis	6 (12.77%)	4 (8.51%)	
CIN III	4 (8.51%)	3 (6.38%)	
Ovarian cyst	10 (21.28%)	6 (12.77%)	
**Type of operation, n (%)**			
Myomectomy	6 (12.77%)	8 (17.02%)	0.387^γ^
Panhysterectomy	30 (63.83%)	33 (70.21%)	
Bilateral adnexectomy	11 (23.40%)	6 (12.77%)	
Preoperative QoR-40 score, mean ± sd	188.21 ± 3.05	188.06 ± 3.05	0.813^α^
Preoperative HR, [IQR]	76 [70, 80]	77 [69, 80]	0.725
Preoperative MAP, [IQR]	88 [85, 93]	85 [80, 89]	0.058

^α^*T*-test.

^β^Mann-Whitney *U*-test.

^γ^Fisher exact test. SD, standard deviation; BMI, body mass index; CIN, Cervical intraepithelial neoplasia; ASA, American Society of Anesthesiologists; QoR-40, 40-item Quality of Recovery Questionnaire; HR, heart rate; MAP, mean arterial pressure.

### 3.2 Primary outcomes

**FIGURE 3:**
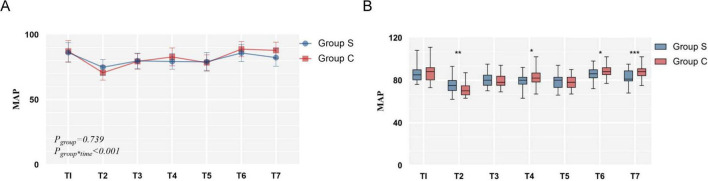
**(A)** The QoR-40 score of the Group S and Group C; **(B)** the differences of five dimensions of QoR-40.

Compared with the Group C, the QoR-40 score of the Group S was higher 24 h after surgery (Group C vs. Group S: 167.47 ± 4.44 vs. 180.13 ± 3.41, *p* < 0.001) (Figure 3A). Among the five sub-items of QoR-40, there were statistically significant differences in the scores of emotional state, physical comfort, psychological support, self-care ability and pain (*p* < 0.001) (Table 2). Compared with the Group C, the Group S showed significant improvement in the above five domains. The differences of five dimensions of QoR-40 between the two groups at 24 h after surgery were visualized by radar chart (Figure 3B).

**TABLE 2:** The comparison of QoR-40 scores.

	Group S (*n* = 47)	Group C (*n* = 47)	*P-*value
Preoperative QoR-40 score, mean ± sd	180.13 ± 3.41	167.47 ± 4.44	<0.001^α^
Emotional state, [IQR]	40 [39, 40]	37 [36, 39]	<0.001
Physical comfort, mean ± sd	50.87 ± 2.25	46.47 ± 2.34	<0.001
Psychological support, [IQR]	33 [33, 34]	31 [31, 32]	<0.001
Self-care ability, [IQR]	24 [23, 24]	23 [23, 24]	<0.001
Pain, [IQR]	34 [34, 35]	29 [28, 30]	<0.001

^α^Analysis of covariance. IQR, Interquartile range; SD, standard deviation; QoR-40, 40-item Quality of Recovery Questionnaire.

### 3.3 Secondary outcomes

#### 3.3.1 The comparison of perioperative HR and MAP

**FIGURE 4:**
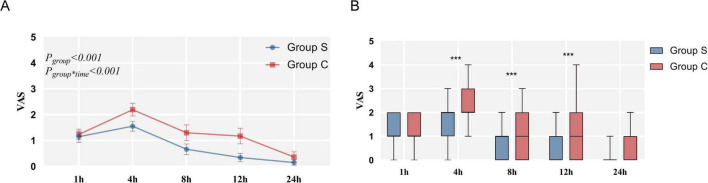
The comparison of perioperative heart rate.

After linear mixed-effects model analysis, there were statistically significant differences in the main effects and interaction effects of perioperative HR intervention factors between the two groups (P_*group*_ = 0.011, P_*time*^*group_<0.001). The individual effects of the intervention at each time point were then analyzed, and significant differences were found between the two groups at T3, T5 and T6 (*P* < 0.001, *P* < 0.001, *P* < 0.001) (Table 3 and Figure 4).

**TABLE 3:** The comparison of perioperative heart rate.

	Group S (*n* = 47)	Group C (*n* = 47)	*P-*value^α^
T1 (bpm), [IQR]	73 [70, 80]	75 [70, 80]	0.756
T2 (bpm), [IQR]	65 [60, 70]	64 [60, 69]	0.609
T3 (bpm), [IQR]	72 [69, 78]	79 [74, 84]	<0.001
T4 (bpm), mean ± sd	66.30 ± 5.60	65.83 ± 5.65	0.728
T5 (bpm), mean ± sd	63.15 ± 5.79	67.47 ± 6.19	<0.001
T6 (bpm), [IQR]	75 [73, 78]	85 [80, 87]	<0.001
T7 (bpm), [IQR]	70 [68, 77]	72 [70, 76]	0.664

^α^Linear mixed-effects model. IQR, Interquartile range; SD, standard deviation; HR, heart rate.

#### 3.3.2 The comparison of perioperative mean arterial pressure

**FIGURE 5:**
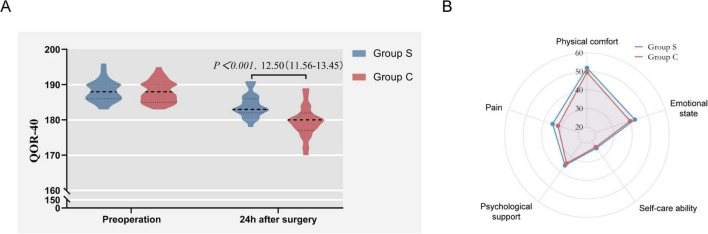
The comparison of perioperative mean arterial pressure.

There were statistically significant differences in the main effects and interaction effects of perioperative MAP intervention factors between the two groups (*P_*group*_* = 0.739, P_*time*^*group_< 0.001). The MAP decreased significantly in the Group S compared with the Group C at T2, T4, T6 and T7 (*P* = 0.002, *P* = 0.019, *P* = 0.023, *P* < 0.001) (Table 4 and Figure 5).

**TABLE 4:** The comparison of perioperative mean arterial pressure.

	Group S (*n* = 47)	Group C (*n* = 47)	*P-*value^α^
T1 (mmHg), [IQR]	85 [81, 89]	88 [80, 92]	0.539
T2 (mmHg), [IQR]	75 [70, 79]	70 [66, 75]	0.002
T3 (mmHg), [IQR]	80 [75, 85]	78 [76, 83]	0.738
T4 (mmHg), mean ± sd	79.38 ± 5.94	82.85 ± 6.81	0.019
T5 (mmHg), mean ± sd	79.04 ± 7.09	78.43 ± 5.96	0.614
T6 (mmHg), mean ± sd	85.83 ± 6.43	88.83 ± 5.81	0.023
T7 (mmHg), [IQR]	81 [80, 88]	88 [84, 91]	<0.001

^α^Linear mixed-effects model. IQR, Interquartile range; SD, standard deviation; MAP, mean arterial pressure.

#### 3.3.3 The comparison of perioperative pain and gastrointestinal function

**FIGURE 6:**
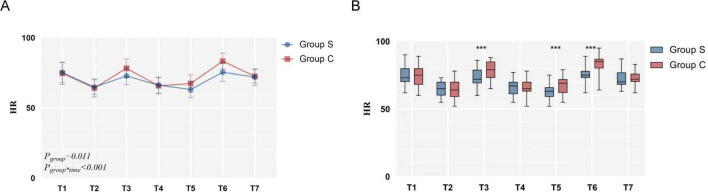
The postoperative VAS score.

Postoperative resting pain was measured by VAS after surgery. Compared to the Group C, the VAS scores were significantly lower at 4, 8, and 12 h after surgery in the Group S (*P* < 0.001, *P* = 0.001, *P* < 0.001). No significant differences in the VAS scores were observed at 1 and 24 h after surgery (*P* = 0.578, *P* = 0.057) (Table 5). However, the number of PCA administrations and the dosage of sufentanil did not differ between the two groups at 24 h after surgery (*P* = 0.069, *P* = 0.985) (Table 6 and Figure 6). SGB can accelerate the recovery of postoperative gastrointestinal function, which is mainly manifested as the shortening of the first anal exhaust time and the recovery time of bowel sounds, and the reduction of the incidence of abdominal distension (*P < 0*.001, *P* < 0.001, *P* = 0.001).

**TABLE 5:** The postoperative VAS score and analgesic drug use.

	Group S (*n* = 47)	Group C (*n* = 47)	*P-*value
Postoperative 1 h VAS, [IQR]	1 [1, 2]	1 [1, 2]	0.578*
Postoperative 4 h VAS, [IQR]	2 [1, 2]	2 [2, 3]	<0.001*
Postoperative 8 h VAS, [IQR]	1 [0, 1]	1 [0, 2]	0.001*
Postoperative 12 h VAS, [IQR]	0 [0, 1]	1 [0, 2]	<0.001*
Postoperative 24 h VAS, [IQR]	0 [0, 0]	0 [0, 0]	0.057*
Postoperative 24 h PCA usage count, [IQR]	0 [0, 0]	0 [0,1]	0.069^β^
Postoperative 24 h sufentanil (ug), [IQR]	60 [55.2, 66.5]	59 [54.2, 67.2]	0.985^β^

*Linear mixed-effects model;

^β^Mann-Whitney *U*-test. IQR, Interquartile range.

**TABLE 6:** The comparison of postoperative gastrointestinal function recovery between the two groups.

	Group S (*n* = 47)	Group C (*n* = 47)	*P-*value
Time to first anal gas passage (h), mean ± sd	22.10 ± 4.24	30.03 ± 3.74	<0.001^α^
Time to first bowel sound recovery (h), mean ± sd	16.1 [13.15, 17.23]	21 [19.86, 23.65]	<0.001^β^
Incidence of abdominal distension n (%)	10 (21.28%)	25 (53.19%)	0.001

^α^*t*-test.

^β^Mann-Whitney *U*-test. IQR, Interquartile range; SD, standard deviation.

#### 3.3.4 Postoperative adverse reactions

Compared with the Group C, the incidence of postoperative nausea, vomiting, headache, shoulder pain and sore throat in the Group S were lower (*P* < 0.001, *P* < 0.001, *P* = 0.014, *P* = 0.006, *P* = 0.006) (Table 7).

**TABLE 7:** The comparison of postoperative adverse reactions between the two groups.

	Group S (*n* = 47)	Group C (*n* = 47)	*P*-value
Nausea n (%)	9 (19.15%)	29 (61.70%)	<0.001
Vomit n (%)	2 (4.26%)	15 (31.92%)	<0.001
Headache n (%)	1 (2.13%)	8 (17.02%)	0.014
Omalgia n (%)	7 (14.89%)	19 (40.43%)	0.006
Pharyngalgia n (%)	5 (10.64%)	16 (34.04%)	0.006

Number of cases (percentage) expressed as n (%).

## 4 Discussion

With the advent of an aging society, the number of perimenopausal women is showing a significant upward trend. Perimenopause is a transitional midlife stage experienced by women. Globally, there are 850 million women aged 40-60, of whom approximately 88% will go through perimenopause. Perimenopausal syndrome is a collection of symptoms related to autonomic nervous system dysfunction and endocrine imbalance. It is primarily caused by hormonal fluctuations and declines in women before and after menopause, and is most commonly observed in middle-aged women aged 45-55 (17–19). Relevant surveys indicate that the neurological symptoms during perimenopause suggest disruptions in various estrogen-regulated systems, including thermoregulation, sleep, circadian rhythm, and sensory processing. While 32.8% of perimenopausal women regain balance through neuroendocrine regulation mechanisms, the remaining women develop perimenopausal syndrome symptoms due to declining hormone levels. These symptoms primarily manifest as psychological, endocrine, and metabolic changes. Furthermore, perimenopausal women often suffer from sleep disorders such as insomnia and disrupted rhythms, which can persist into late menopause. These disorders are accompanied by symptoms such as vasomotor disturbances, anxiety, depression, and cognitive decline, all of which negatively affect quality of life (20–22). The psychological wellbeing of women with perimenopausal syndrome is generally poor, making it a societal issue that imposes certain burdens on families and communities. Additionally, the physical and psychological stress from surgery and anesthesia further impacts the postoperative recovery of perimenopausal women. According to a 2005 study by the U.S. National Institutes of Health, approximately 39–47% of perimenopausal women experience sleep disorders, with the proportion rising to as high as 60% among menopausal women (23).

With the continuous advancement of laparoscopic technology, gynecological laparoscopic surgery has become a widely used and advanced alternative to traditional open surgery. Compared to open surgery, laparoscopic procedures offer several advantages, including minimal trauma, faster recovery, and fewer complications. For female patients in particular, laparoscopic surgery significantly reduces incision size and scarring, which is a major benefit for those who value aesthetics. As a result, laparoscopic surgery has gained widespread popularity among patients in clinical practice (24, 25). A study by Medeiros et al. (5) involving 324 women with benign ovarian tumors demonstrated that compared to open surgery, laparoscopic surgery reduced the incidence of fever, urinary tract infections, postoperative complications, postoperative pain, hospital stay duration, and overall costs. Related research (26) also showed that for the treatment of gastric cancer, laparoscopic surgery not only reduced postoperative complications but also had less impact on patients’ humoral and cellular immune functions, thereby promoting recovery. Consequently, laparoscopic surgery has been adopted in major hospitals. While laparoscopic surgery has its unique advantages, it also presents challenges that raise concerns. The establishment of CO_2_ pneumoperitoneum and the adjustment of special intraoperative positions can lead to a series of adverse effects, potentially impacting the patient’s postoperative recovery.

To ensure a clear surgical field, separate organs from the abdominal cavity, and appropriately expand the abdominal space for better visualization of abdominal structures, CO_2_ is introduced into the abdominal cavity during laparoscopic surgery, establishing an artificial pneumoperitoneum. However, this process also brings related effects. Impact of CO_2_ on the respiratory system: The increased intra-abdominal pressure caused by CO_2_ pneumoperitoneum elevates the diaphragm and reduces lung compliance. This is especially pronounced in gynecological laparoscopic surgery, where patients are positioned in a Trendelenburg position (head down, feet up), leading to increased physiological dead space and ventilation-perfusion mismatch. Impact of CO_2_ on the circulatory system: The high solubility of CO_2_ and the pressure gradient between the abdominal cavity and blood can lead to rapid CO_2_ absorption. Prolonged laparoscopic procedures may result in hypercapnia and acidosis, indirectly stimulating aortic chemoreceptors and carotid sinus receptors. This increases the concentrations of plasma catecholamines, cortisol, and vasopressin (6, 27). Elevated intra-abdominal pressure can cause visceral vasoconstriction and reduced blood flow in major veins such as the inferior vena cava, renal veins, and hepatic veins, thereby decreasing cardiac preload. Laparoscopic surgery can also disrupt the autonomic nervous system’s balance between sympathetic and parasympathetic activity. This imbalance may lead to unexpected increases in mean arterial pressure, heart rate, and systemic vascular resistance, while reducing cardiac output. Consequently, conditions such as hypertension, arrhythmias, tachycardia, and bradycardia may occur (28). Female patients undergoing surgery have a high incidence of postoperative nausea and vomiting (PONV), and the effects of pneumoperitoneum further increase this risk. Previous studies have reported that the postoperative symptom rates for patients undergoing laparoscopic surgery are approximately as follows: 45% for pain, 17% for nausea, 8% for vomiting, 17% for headache, 42% for drowsiness, 18% for dizziness, and 21% for fatigue (29). These symptoms significantly impact the postoperative recovery of patients undergoing laparoscopic surgery.

The stellate ganglion is a fusion of the 6th and 7th cervical ganglia with the 1st thoracic ganglion, and it is one of the primary sympathetic ganglia in the human body, hence also called the cervical stellate ganglion. Stellate ganglion block (SGB) is one of the oldest and most commonly performed sympathetic nerve blocks. SGB is widely applied in clinical practice and extensively studied in terms of its mechanisms and clinical uses. By injecting local anesthetics into the cervical stellate ganglion and the surrounding loose connective tissue, voltage-gated sodium ion channels on the neuronal cell membrane are blocked (30). Its mechanism of action mainly involves the central and peripheral nervous systems (31). In the central nervous system, it stabilizes the internal environment by regulating hypothalamic activity. In the peripheral nervous system, it blocks preganglionic and postganglionic fibers, thereby inhibiting the sympathetic nervous system in the corresponding innervation regions, achieving therapeutic effects for related diseases. Studies have shown that left-side SGB can treat ischemic ventricular arrhythmias (32). The left stellate ganglion is highly involved in regulating ventricular electrophysiology. Excessive sympathetic nerve activity in the myocardium can lead to ischemic ventricular arrhythmias. Left SGB can impair left ventricular function and reduce stroke volume (33). In contrast, right-side SGB modulates the balance between sympathetic and parasympathetic activity with minimal impact on circulation (34). Additionally, right-side SGB improves the oxygen supply-demand balance in coronary arteries during acute coronary occlusion, reducing the risk of myocardial ischemia (35). Right SGB also helps stabilize cardiac electrical activity (36). Animal studies have indicated that right-side SGB can improve spatial learning and memory impairments in sleep-deprived rats and increase serum melatonin levels (37). Therefore, this clinical study selects right-side stellate ganglion block for investigation.

With the continuous advancement of medical technology, the clinical application of ultrasound has become increasingly widespread. Ultrasound-guided nerve puncture techniques are now well-known to anesthesiologists. Ultrasound guidance allows for a more intuitive understanding of the structures near nerves, such as tissues and blood vessels, thereby reducing related complications such as hematoma, hoarseness, and difficulty swallowing. Additionally, effective nerve blockade can be achieved with smaller doses of local anesthetics, reducing their usage and enhancing procedural safety (38). Ropivacaine, a commonly used local anesthetic, belongs to the long-acting amide class and has low toxicity to the central nervous system and heart (39). Research by Gul Jung and colleagues demonstrated that using 4 mL of 0.2% ropivacaine for right stellate ganglion block (SGB) achieved optimal blockade effects with high safety (40). Therefore, this study employed ultrasound-guided puncture with 4 mL of 0.2% ropivacaine. Patients in the S group all exhibited Horner’s syndrome after the block without adverse reactions, further confirming the safety and efficacy of this blocking protocol. A study by Hai-Hua Shan et al., involving 130 patients, found that under ultrasound guidance, the modified puncture at the level of the C6 transverse process (C6 group) required less time and fewer angle adjustments compared to puncture at the C7 transverse process (C7 group). Additionally, the incidence of adverse reactions was lower in the C6 group. Based on these findings, this study adopted the modified C6 transverse process-level puncture technique. The puncture process was smooth, the patients reported a good experience, and no significant adverse reactions were observed, further demonstrating the practicality and feasibility of this puncture method.

The concept of ERAS (Enhanced Recovery After Surgery) was initially referred to as “fast track surgery” and was first introduced in 1994 by Engelman and colleagues in the field of cardiac surgery (41). ERAS not only refers to the reduction in hospital stay but also aims to normalize physiological function during the perioperative period through evidence-based measures, thus minimizing surgical complications and promoting accelerated recovery post-surgery. In clinical practice, anesthesiologists are continuously exploring ERAS methods to improve postoperative recovery quality and increase patient satisfaction. The primary outcome measure of this study was postoperative recovery quality, assessed using the Chinese version of the QoR-40 scale to evaluate recovery within 24 h post-surgery. The QoR-40 scale includes 40 items covering five aspects: physical comfort, emotional state, self-care ability, psychological support, and pain, with a maximum score of 200 points (42). QoR-40 has high practical utility and reliability for assessing early postoperative life and recovery quality and is widely used in clinical research (43). In this study, the QoR-40 questionnaire was used to evaluate the postoperative recovery quality of perimenopausal patients undergoing laparoscopic surgery. The results showed that the total QoR-40 score at 24 h post-surgery for Group S (180.13 ± 3.41) was significantly higher than Group C (167.47 ± 4.44) (*P < 0.001*), indicating that right-sided SGB before surgery can improve postoperative recovery in perimenopausal patients undergoing elective laparoscopic surgery. Among the five subscales, significant differences were observed in emotional state, physical comfort, psychological support, self-care ability, and pain (*P* < 0.001). When considering the incidence of postoperative adverse reactions (nausea, vomiting, headache, shoulder pain, and throat pain), the S group had lower rates than the C group (*P* < 0.001, *P* < 0.001, *P* = 0.014, *P* = 0.006, *P* = 0.006), suggesting that preoperative SGB can reduce related adverse reactions, alleviate postoperative pain, and thus improve postoperative comfort and self-care ability. This, in turn, enhances the emotional state and physical comfort of the patients, overall improving postoperative recovery in perimenopausal patients.

Postoperative sleep quality is also an important component of ERAS. Better sleep quality promotes recovery, and clinical anesthesiologists have been seeking methods to improve postoperative sleep quality. Recent clinical studies have shown that SGB can improve postoperative sleep quality and alleviate sleep disturbance (44, 45). In this study, Group S had higher scores for physical comfort and emotional state than Group C, which may be due to the improvement in postoperative sleep quality by SGB, thereby increasing these two scores.

Postoperative pain is also a major concern for anesthesiologists. Improper management of postoperative pain, if not addressed promptly, can result in the development of acute pain into chronic pain (46). SGB has been widely used for various sympathetic nerve-mediated pains (47), with the goal of interrupting the pain cycle by blocking sympathetic nerves and restoring normal somatic sensory balance through the recovery of the pain area (48). A linear mixed-effects model was selected to analyze the changes in postoperative resting pain scores, based on two considerations: (1) the data did not follow a normal distribution and the follow-up time points were not equally spaced; (2) some data might be missing due to nighttime sleep during the postoperative follow-up at 4, 8, and 12 h. The mixed-effects model is suitable for analyzing such panel data characteristics. The results of this statistical analysis showed that the resting pain scores at 4, 8, and 12 h post-surgery were lower in Group S compared to Group C (*p* < 0.001, *P* = 0.001, *P* < 0.001). This indicates that preoperative SGB can improve postoperative pain, which is consistent with previous research (10, 49). Our study results showed no differences in the resting pain scores, PCA frequency, and sufentanil usage at 24 h post-surgery between the two groups. This suggests that preoperative SGB can improve early analgesic effects (within 12 h post-surgery). Related research indicates that preoperative SGB can effectively suppress stress responses during anesthesia, reduce blood pressure fluctuations during anesthesia induction and intubation, decrease blood catecholamines during CO_2_ pneumoperitoneum, and maintain hemodynamic stability during the perioperative period (50, 51). The HR and MAP trends for both groups show that Group S had less fluctuation and more stable hemodynamics than Group C, which is consistent with prior studies.

The recovery of gastrointestinal function after surgery is also an important concern for both clinicians and patients. Relevant studies have shown that SGB can promote the recovery of gastrointestinal function postoperatively (52). This study shows that, compared to Group C, Group S had a significantly shorter time to first anal flatus by 7.93 h (6.27-9.58) (mean difference and 95% CI) (*P* < 0.001), and a shorter time to the return of bowel sounds by 5.23 h (3.98-7.45) (median difference and 95% CI) (*P* < 0.001). In Group S, 10 cases of postoperative bloating occurred, with an incidence of 21.28%, while in Group C, 25 cases of postoperative bloating occurred, with an incidence of 53.19% (*P* = 0.001). These results indicate that preoperative SGB can also promote the recovery of gastrointestinal function after laparoscopic surgery in peri-menopausal women. The possible mechanism is that SGB regulates the balance between the sympathetic and parasympathetic nervous systems, making the parasympathetic nervous system relatively more active, which in turn promotes gastrointestinal motility and facilitates the recovery of gastrointestinal function (53).

Limitations and shortcomings of this study: (1) Considering the ethical principle of non-maleficence, the study did not include a placebo-controlled group in Group C, which may have influenced the results. (2) This study is a single-center, small-sample study. Future multi-center, large-sample clinical studies are needed to provide more evidence on the safety and effectiveness of SGB in peri-menopausal women undergoing gynecological laparoscopic surgery.

## 5 Conclusion

Preoperative SGB can improve postoperative recovery quality in peri-menopausal women undergoing gynecological laparoscopic surgery, effectively relieve early postoperative pain, stabilize perioperative hemodynamics, promote the recovery of gastrointestinal function, and reduce the occurrence of postoperative adverse reactions.

## Data Availability

Publicly available datasets were analyzed in this study. This data can be found here: the Ethics Committee of Fujian Provincial Hospital The original data can be acquired by connecting to the corresponding authors.

## Footnotes

1
www.chictr.org.cn
